# The Tendency of Terrorist Organizations to Explosive Attacks: An Institutional Theory Perspective

**DOI:** 10.3389/fpsyg.2022.747967

**Published:** 2022-02-16

**Authors:** Lanjun Luo, Chao Qi

**Affiliations:** School of Management, Huazhong University of Science and Technology, Wuhan, China

**Keywords:** explosive attack, institutional theory, organizational isomorphism, terrorist organization behavior, terrorism contagious

## Abstract

Focusing on the tendency of terrorist organizations to explosive attack, this article applied the institutional theory as the basis to explain the inherent logic of attack type similarity from the perspective of mimetic, coercive, and normative isomorphism. Subsequently, the study conducted an empirical analysis of the data onto 1825 terrorist organizations recorded in the Global Terrorism Database with the logistic regression method. The results show that: (1) Terrorist organizations will learn from pre-existing terrorist organizations' experiences, and mimetic isomorphism will promote explosive tendency; (2) Due to the normative isomorphism effect, terrorist groups' tendency to explosive attacks is weakened by their increased duration; (3) If terrorist organizations are hostile to a strong government, coercive isomorphism positively moderates the negative effects of increasing duration. The study suggests that counter-terrorism approaches such as destroying the learnable experience of attacks, addressing the root causes of terrorism, and maintaining a strong government may be helpful in stopping increasing terrorist activities, which is essential for reducing terrorist organizations' vivosphere, blocking the inter-flow and imitation between terrorist organizations, and ultimately interrupting the terrorist propagation chain.

## 1. Introduction

**Figure 1:**
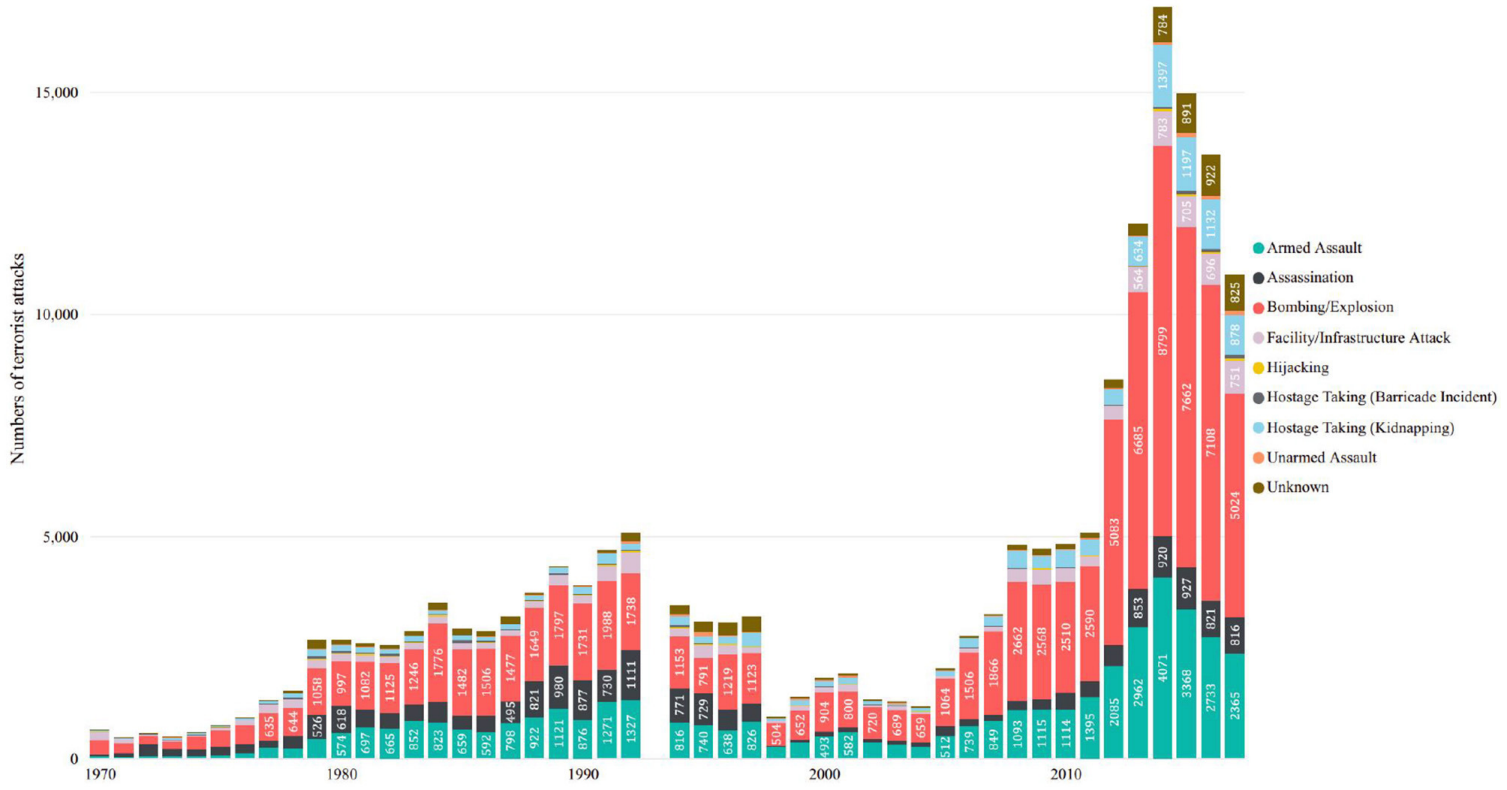
Global terrorism trend and proportion of different attack types (Data source: GTD, 1970-2017, attack type statistics using GTD's variable *attacktype*1_*txt*).

After 9/11, counter-terrorism (CT) became a priority for homeland security worldwide, and enormous amounts of material and human resources are cost in this area. However, many intelligence-led CT and prevention approaches, regarded as pre-emptive and precision strikes, have not achieved the desired results but instead have added fuel to the fire (Bjørgo, 2013). As shown in Figure 1, the frequency of terrorist attacks worldwide, as recorded in the Global Terrorism Database (GTD) (LaFree and Dugan, 2007; START, 2021), is still on an upward trajectory. This unexpected trend means that CT operations have not stopped the actions of terrorists and terrorist organizations, the adversarial strategies still have not entirely grasped the behavioral patterns of enemies. Therefore, to make CT more effective, a better understanding of the behavioral patterns of terrorist organizations and the underlying causes is necessary.

**Figure 2:**
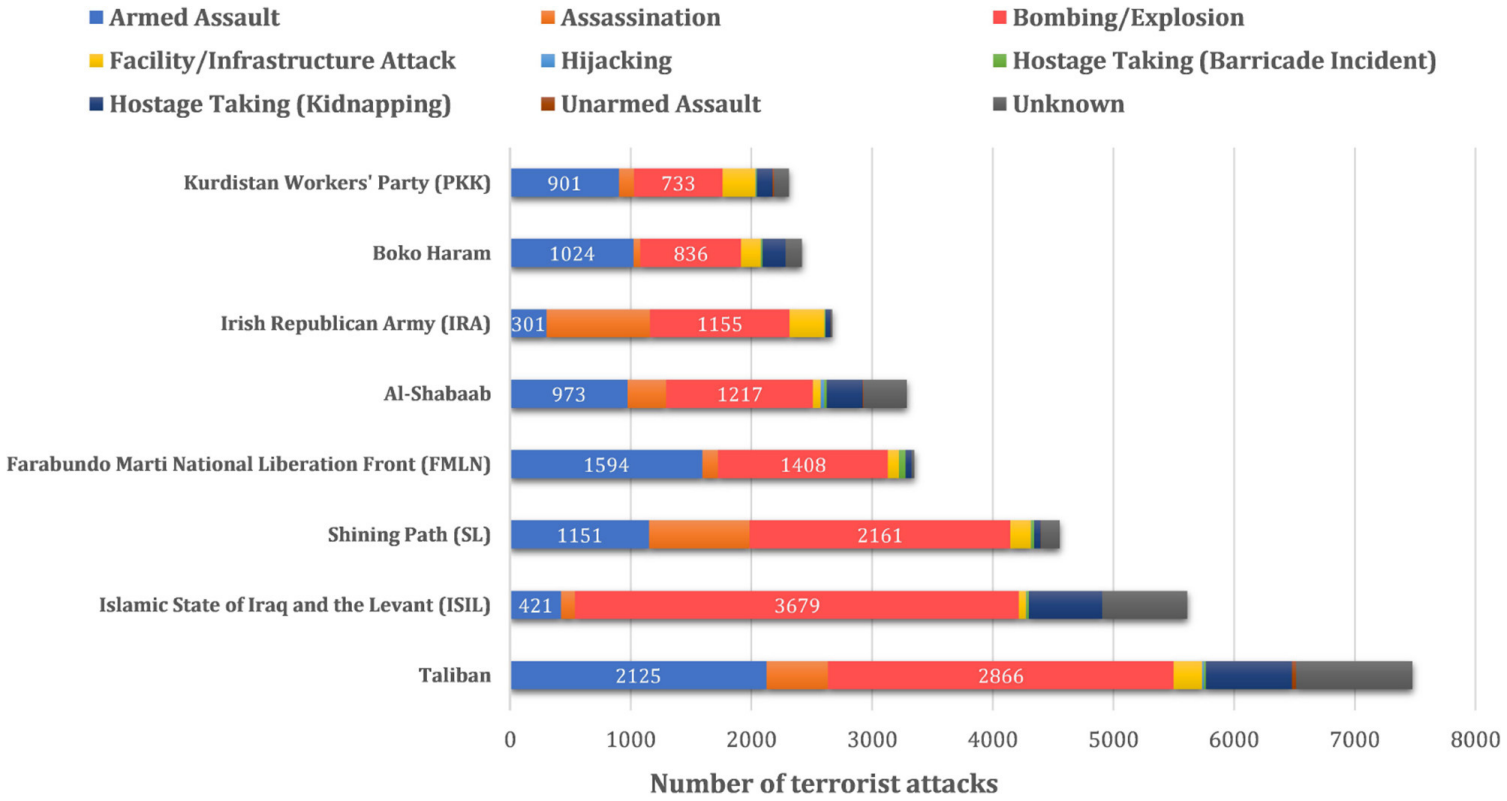
Comparison of several terrorist organizations' attack methods (Data source: GTD, 1970–2017, the total attack number of a specific terrorist organization is calculated by the times recorded in GTD's variable *gname*, while the attack types statistics by *attacktype*1_*txt*).

One of the most critical aspects of terrorist organization behavior patterns is the attack type. As shown in Figure 1, the explosive attack is the most used type worldwide each year. This article has also counted the attack types of 181,691 cases in GTD, and the results are shown in Table 1. The most frequent attack type is still bombing and explosion, with 88,255 records and accounting for 48.57% of the total, far more than any other terrorist attack category. Moreover, different terrorist organizations also demonstrate a tendency to commit explosive attacks. Figure 2 illustrates an analysis of the attack types of several notorious terrorist organizations. It can be seen, these terrorist organizations have shown a focus on explosive attacks, e.g., both the Taliban and the Islamic State of Iraq and the Levant (ISIL) use explosions as the most common method. For organizations such as the Farabundo Martí National Liberation Front (FMLN), explosive attacks, while not the most frequent, are still among the most common.

**Table 1:** The number of different attack types in GTD (Data source: GTD, 1970-2017, attack type statistics using GTD's variable *attacktype*1_*txt*).

**Method of terrorist attacks**	**Number of cases**
Bombing/Explosion	88,255
Armed assault	42,669
Assassination	19,312
Hostage taking (Kidnapping)	11,158
Facility/Infrastructure attack	10,356
Unknown	7,276
Unarmed assault	1,015
Hijacking	991
Hostage taking (Barricade incident)	659

Why do terrorist organizations have a similar behavior pattern that tends to explosive attacks? To our knowledge, few studies directly answer this question, but some of them address other aspects of terrorist organization similarity. Representative, Di Salvatore (2018) found pirates similarly tend to cluster in places where successful attacks have occurred. Parker and Sitter (2016) found terrorist organizations with similar ideologies were similar in tactics, strategies, and objectives. Both pieces of research regard the similarity of terrorists' behavior patterns as mainly a result of inter-organization learning and imitating process, while the latter keenly recognized the process is analogous to the isomorphism of enterprise organizations analyzed by institutional theory (DiMaggio and Powell, 1983) in sociology.

However, the existing approaches are not sufficient to explain the explosive tendency. Firstly, in terms of explosive attacks, the objects of different terrorist organizations imitating and learning from are unclear. Most terrorist organizations mainly operate in a limited area or within a relatively fixed range. For example, Abu Sayyaf primarily operates in Chechnya and Laskhar Jihad in Indonesia (Ganor, 2008). Even for transnational or international terrorist organizations, the preparation and requirements for launching explosive attacks in different countries are heterogeneous. How do terrorist organizations in different areas choose the imitation targets and learn from them? Secondly, the formation of similar behavioral patterns in terrorist organizations is not only free will-led learning results but may be shaped by complicated conditions and environments. In Western or well-policed societies terrorists are heavily repressed, and less restricted in lawless areas (Ganor, 2008). Terrorists are not at liberty to learn from others' experiences and practice them. CT forces are constantly trying various military, political, and economic methods to forge an “iron cage” in the hope of hanging them. How does external coercion shape the similarity of terrorist organizations? Thirdly, if terrorist organizations can survive under coercion for a longer time, then it seems they can choose other more destructive and specialized attacks to fulfill their political or social goals. For example, plane hijackings always create serious media attention and chaos (Terrorism-Research.com, 2021). How do the increasing professionalization and operational capacity represented by a terrorist organization's persistence affect organizational similarity?

The complete institutional theory may help answer the above questions, analyzing how multiple driving forces shape organizational similarities is its core (DiMaggio and Powell, 1983). The organizations' isomorphism phenomenon, which manifested in the resemble of different organizations, is caused by three types of isomorphism: mimetic isomorphism due to dealing with uncertainty, coercive isomorphism driven by legal or political pressure, and normative isomorphism due to professional experts and organizations (DiMaggio and Powell, 1983; Hersberger-Langloh et al., 2021). Early institutional theory studies have focused primarily on the isomorphism of typical organizational forms and practices (Ufere et al., 2020). Recently, more research has paid attention to illegal behaviors, e.g., the bribery pervasive among firms (Ufere et al., 2020), and the criminogenic isomorphism in the business context (Glebovskiy, 2019). There are also studies that apply institution theory to the public safety issues, such as the widely social media adopting of police departments (Hu et al., 2018), and the similarities in investigating human trafficking law enforcement efforts (Warren, 2019).

The three isomorphic processes in institutional theory have focused on the causes and targets of organizational learning, the influence of external pressures, and the impact of professionalization. Such findings may help us understand the learning reason and objects of terrorist organizations, the effects of CT pressures, and the influence of capability and specialization represented by the age of the organization. Nevertheless, many internal isomorphism mechanisms are still puzzling, and the relevant studies of institutional theory have not delved into the issue of terrorist organization behavioral isomorphism, especially the empirical research on the tendency to explosive attacks is still a gap.

Therefore, this study attempts to develop a conceptual model to explain the terrorist organizations' explosive attack tendency from the perspective of institutional theory, and to analyze the behavioral similarity of terrorist organizations based on the three drivers in institutional theory—coercive, normative, and mimetic isomorphism. In particular, we aim to answer the following three research questions in an extension of existing terrorism researches and institutional theory studies:

- How do terrorist organizations mimic explosive attack experiences, and who are the learning targets?
- How does the increase of the terrorist organizations' persistence affect their explosive attack tendency?
- How do CT pressures shape terrorist organizations' explosive tendency?

The contributions of this article are mainly threefold. Firstly, we apply institutional theory to the analysis on the illegal behavioral isomorphism of terrorist organizations; previous studies have mainly been limited to legal organizations' legal or illegal behavior, which facilitates the application of institutional theory. Second, based on the institutional theory's mimetic isomorphic drive, we analyze the possible targets for terrorist organizations to learn from the experience of bombing attacks cross-organisationally. Thirdly, based on normative and coercive isomorphism, the difference in approach between terrorist organizations and ordinary business organizations in the face of institutional iron cages is analyzed, and short- and long-term combined CT policy recommendations are given.

The rest of this study is structured as follows. In section 2, we review the related studies. The main hypotheses of this study are presented in section 3. Section 4 explains the variable selection and data obtaining methods. Logistic regression results are in section 5. In section 6, we discussed this study's management implications and limitations. Finally, the research is concluded in section 7.

## 2. Literature Review

This section first reviews the common organizational isomorphism phenomenons in the institutional theory researches and how the three structures of uncertainty-mimetic isomorphism, professional-normative isomorphism, and formal pressure-coercion isomorphism come into play. Second, we introduced other alternative terrorist attack types and the differences with explosive attacks. Third, the related institutional environments and root causes of terrorism are reviewed.

### 2.1. Institutional Theory and Its Applications

Institution, defined as “regulative, normative, and cognitive structures and activities that provide stability and meaning for social behavior” (Scott and others, 1995). As a crucial part of the whole environment, institutions include laws, social and professional norms, culture, ethics, etc. (Miles, 2012). The institutional theory studies mainly focus on how the organizations evolve toward similarity in the institutional environment. The three isomorphic mechanisms proposed (DiMaggio and Powell, 1983) have become the mainstream tools of this research field.

Existing institutional theory researches can be divided into three categories depending on the research objectives. First, the most orthodox and mainstream application of institutional theory is to explain legitimate business organizations' legal behavior and structural isomorphism. Davis et al. (2000) applied the institutional theory framework to analyze the international entry mode of strategic business units. Brouthers et al. (2005) theorized that emerging market firms can improve export performance satisfaction by imitating the modal generic product strategy from the home country multi-national enterprises. Marquis et al. (2007) found the institutional pressures at the community level can make corporate overcome immediate profit maximization goals and focus more on social benefits and issues. Zach et al. (2021) found determinants such as alliance membership and firm size play an important role in innovation copying, an inter-firm mimetic isomorphism phenomenon.

Second, some studies focus on the non-legitimate behavior isomorphism of legitimate business organizations. Venard (2009) found competitive and institutional isomorphism have a crucial influence on the corruption in Russia firms. Among the three isomorphic drives, the normative isomorphism (imitation to competing firms) and the mimetic isomorphism (ethical behavior brought by multi-nationals) both have a significant effect on corrupt behavior in Russian firms, while the coercive isomorphism (quality of the legal framework, law, and financial markets) was shown not applicable. Gao (2010) found that the mimetic isomorphism motivated by other firms' bribing behavior significantly affects the bribery of firms in transitional China. Ufere et al. (2020) found the mimetic isomorphism (perception of frequently bribery practices in specific industries) and the coercive isomorphism (institutional constraints on businesses) have positive relationships with bribery pervasive among sub-Saharan African firms.

In addition, police organization behavior and structure isomorphism is also a theme. Giblin (2006) proofed the institutional factor—accreditation, can represent both normative and coercive pressure, while increases the isomorphism odds of the crime analysis unit incorporation in police organizations. Soeters (2008) found common experiences and inter-military learning shape the operation isomorphism of American and Netherlands armed forces in Afghanistan. Obviously, mimetic isomorphism played an important role. Dupont (2015) assessed the limitations of CT forces to generate an adversarial isomorphic network structure against terrorists. This comes from the observation that new terrorists are similar in building a structure of decentralized distributed networks, thus gaining more ability to sustain CT strikes. Burruss and Giblin (2014) applied institutional theory to analysis the similar adoption of community policing in municipal law enforcement agencies. They found centrist forces, e.g., professionalization of law enforcement, are the driver of the community policing isomorphism. Carter (2016) found the institutional pressures have a significant positive effect on the adoption of intelligence-led policing.

In general, institutional theory has been well applied to explain the isomorphism of legal and illegal behavior in various kinds of legitimate organizations. Existing researches also help us understand how the three isomorphic processes operate. The research on illegal behavior and policing organizations' similarities lead us to believe that this theory can also be applied to the isomorphism of terrorist organizations. However, the analysis of the co-occurrence of illegal organizations and illegal behaviors remains missing in the existing studies.

### 2.2. Terrorism, Explosive Attack, and Alternative Types

When the common tendency of terrorist organizations to explosive attacks is found, it is necessary first to review what terrorism is and what other attack types besides bombing are available.

Terrorism, as a complex aggregate of concepts and phenomena, is still difficult to define in a thoroughly uniform single manner (McCann and Pimley, 2020). The relatively commonly accepted definition is that terrorism is an act of a political, violent, radical nature involving non-state actors with the intention of spreading a broader psychological fear effect (Byman, 2020). As shown in Table 1, except for the explosive and bombing attack, there are seven alternative attack types: assassination, armed assault, hijacking, two kinds of hostage-taking, facility and infrastructure attack, and unarmed assault. The difference between these methods and explosive attacks needs to be clarified. All the eight types can be divided into three categories according to their objects: against human objects, against material objects, and hybrid.

In GTD, The armed assault attacks aim to cause harm or death to human beings with firearms or other lethal instruments, e.g., the attack against Shia prisoners in June 2014 (Sly and Ramadan, 2014; START, 2021). In comparison, the unarmed assault attacks want to achieve the same goal by using other means, including biological, chemical, and radiological, e.g., the chemical-related incident in Uganda in March 2000 (Cirjak Antonia, 2020; START, 2021). Further, hostage-taking (two kinds) and assassination are apparent human targeted attacks. Unlike the first category, the hijacking with the primary object of taking control of vehicles and the facility-infrastructure aimed are both attacks against material objects. The representative case for the former type is the “9/11” attack, and the latter is the bus attack in Angola in August 2001 (Guardian, 2001; START, 2021).

Unlike other types, explosive attacks' objects are hybrid. Both people and materials can be the targets. For example, the coordinated vehicle explosive attacks in Iraq in August 2007 set the first target on minority people while also destroying villages (START, 2021). In addition to the difference in target, the major difference between explosive and other methods is that bombings are relatively cheap and easy to make and are smaller, harder to detect, and often highly damaging (Terrorism-Research.com, 2021). And Parker and Sitter (2016) also thought the advances in weapon affordability, portability, and concealability gave terrorists a “force multiplier.” Compared with other methods such as kidnapping, which is regarded as “one of the most difficult acts” (Terrorism-Research.com, 2021), explosive seems to serve the needs of terrorists better.

However, although explosives have some advantages in launching attacks, the occurrence of specific attacks is the result of the multiple-coupling conditions and is not entirely the weapon power factors' consequence such as convenience, cheapness, and concealability. In the view of situational crime prevention research, the target constitutes one of the most crucial aspects besides weapon selection (Clarke and Newman, 2006; Hsu and Newman, 2016). Elements such as the target's exposure, vitality, destructibility, and iconicity trigger specific terrorist attacks (Clarke and Newman, 2006). Then, if a terrorist group's experience is all against unarmed or weakly defended targets, perhaps it would hardly be a learning destination for terrorist organizations operating against powerful even military targets. At the same time, the degree of defense is often related to various political, military, and economic factors in the institutional environment where the target is located. Therefore, it is necessary to consider broader environments and examine who the different terrorist organizations can effectively learn from.

### 2.3. The Institutional Environment of Terrorist Organizations

The institutional environments at the country level are usually composed of both formal and informal institutions (Hitt, 2016). This is true for business organizations, and it is also valid for illegal organizations. For firms, the traditional institutions are represented by laws and standards (Scott and others, 1995), and the informal institutions is represented by culture (Fu et al., 2004). For terrorist organizations, the institutional environment is most likely the root cause of terrorism.

Terrorism originates in wide economic, political, and conflict backgrounds, many root cause analyses are exploring “what breeds terrorism” (Kis-Katos et al., 2011). These root causes collectively form the most important part of the institutional environment where terrorist organizations exist. Typically, for economic causes, Krieger and Meierrieks (2019) found that the higher income inequality level caused worse institutional corruption and more domestic terrorist activity. Ajide and Alimi (2021) found income inequality, human capital, and educational attainment have impact on terrorism. Bagchi and Paul (2018) found youth unemployment tends to increase domestic terrorism while may not effect the transnational terrorism. Gassebner and Luechinger (2011) found factors such as GDP, population, and economic freedom have significant impact on terrorism. For political causes, Ajide et al. (2020) found natural resource rents, political regimes (democracy and autocracy) both have effects on terrorism. Baek and Bouzinov (2021) found the increasing effect of democratization on terrorism reaches peak when a country is at very middle democracy level. For conflict, Schumacher and Schraeder (2021) found the domestic political instability (government purges and riots) increase terrorism. Shahzad et al. (2020) found foreign aid fuels terrorism because of the institutional problems and civil conflicts. Gassebner and Luechinger (2011) also investigated the influences on terrorism from guerrilla war, internal war, ethnic tensions, and other conflict factors.

Meanwhile, just as businesses are not prisoners of their institutional environments, but may act in creative ways to change their institutional environments (Dimaggio, 1988), terrorist organizations are in turn influencing theirs. Inspiring, Zulfiqar et al. (2014) found terrorism negatively influenced the foreign direct investment in Pakistan. Shahzad et al. (2019) found terrorism increased the capital flight. Lanouar and Shahzad (2021) found terrorist attacks in big cities can cause the highest negative impact on the capital flow. Evidence of increased capital flight due to terrorist attacks is also further confirmed in the study of Shahzad and Qin (2019). Through the above research, it is clear to see how terrorist organizations and terrorist attacks inversely act on the institutional environment.

Moreover, along with other formal coercive powers, CT activities are also an important part of constructing the institutional environment for terrorist organizations. At the macro level, Shahzad and Qin (2019) found military expenditures can decrease the impact of terrorism on the capital flight. Shahzad et al. (2019) also thought military expenditures, counter terrorism policies, stable political environment, and better economy contribute to the containment effect of curbing the negative effects of terrorism. At the micro level, Di Salvatore (2018) found that for pirates who like to cluster in previous successful locations to repeat their attacks, rescue operations can reduce most attacks in the following month. The similar control benefit diffusion effects may be valid for other terrorist attack types (Hsu and Newman, 2016).

In conclusion, the above reviews reveal the dialectical relationship between terrorist organizations and their institutional environment. In the next section, we will explore how institutions make terrorist organizations become similar in the attack type and how terrorist organizations break the institutional iron cage.

## 3. Research Hypotheses

### 3.1. Mimetic Isomorphism: Limited Imitation Objects

Uncertainty is one of the most critical forces that cause mimetic isomorphism (DiMaggio and Powell, 1983; Miles, 2012). Uncertainty means that the organization does not know what to do because, e.g., ambiguous goals and uncertainty about appropriate behavior (Miles, 2012; Deligonul et al., 2013). Thus, Organizations like firms tend to imitate the successful behavior of others in the same organizational field (Ufere et al., 2020). Although Parker and Sitter (2016) found that the behavior of early terrorists may provide ideological and tactical inspiration for the later ones. However, in terms of specific terrorist organizations wishing to learn the experience of explosive attacks, it remains unclear who these prior organizations were.

The two crucial points—uncertainty and organizational domain—help to answer the question of learning objects. Researches have demonstrated how the root causes of terrorism interact with terrorist attacks at the country level (Gassebner and Luechinger, 2011). The countries that terrorist organizations confront often constitute the most representative organizational field. Terrorist organizations in the same country with similar primary operating areas will most often face similar pressures from CT actions, political penalties, economic sanctions, etc. For example, in Iraq, during the period of US-led coalition intervention, improvised explosive device attacks are always likely leading to similar clustered counter-insurgency activities (Braithwaite and Johnson, 2012). The same organizational field, which is destructive and uncertain to terrorist attacks, has effected not only pre-existing terrorist organizations, but also subsequently arising terrorist organizations. The most immediate response of the newcomers to this pressure is to learn from the experience of other pre-existing terrorist organizations in the same organizational domain, namely the same country. Although it is possible to learn terrorist attack experience through the Internet, the actual bomb-making, target investigation, and other techniques in specific contexts, terrorists can only gain the most intuitive experience by learning in the real world (Bruce, 2009; Kenney, 2009). Rather than learning from other terrorist groups' bombing experiences in countries with different institutional environments, it may be better for a terrorist organization to learn from the behavior of existing groups in the same specific country. In other words, terrorist organizations imitate a limited range of objects, most probably organizations that operate mainly in the same countries. The explosive tendency and experience of existing terrorist organizations will greatly influence the latter's behavior. Thus:

**H1**: Pre-existing terrorist organizations' tendency to explosive attacks may have a positive impact on the tendency of later groups in the same country.

### 3.2. Normative Isomorphism: Professionalization and the Asthenia of Iron Cage

In institutional theory, normative isomorphism is primarily caused by professionalization (DiMaggio and Powell, 1983), standards, employee movement (Miles, 2012), etc. In turn, business organizations become similar under these specialized forces. However, different from firms, the professionalization may fuel terrorist organizations to break the institutional iron cage, rather than conform.

Facing external threats, terrorist organizations often cooperate and train attack experts with each other in order to strengthen themselves and keep their organizations alive (Phillips, 2014). This inter-organizational circulation of personnel points to the professionalization of modern terrorist organizations. Similar to organized criminal groups (Sanderson, 2004), personnel training, equipping, recruiting, transporting, and intelligence acquisition all form part of the normalization and specialization requirements of terrorist organizations. Thus, if a terrorist organization persists for an extended period, it usually accumulates a pool of terrorists with multiple attack capabilities, which potentially strengthens its bargaining power against governments as well.

The longer a terrorist organization lasts, meaning the more the iron cage holding it collapses, the more bargaining power it has, and it may no longer stick to explosive attacks but will consider more alternative approaches. At this time terrorist organizations may be thinking about getting more results, such as terror and disruption. Because of increased professionalization, the attack type is no longer confined to explosions, the affordability, portability, and concealability of weapons are no longer crucial. Thus:

**H2**: The longer duration of a terrorist organization has a negative impact on the explosive attack tendency.

### 3.3. Coercive Isomorphism: Reinforcement of the Iron Cage

Although the professionalization and persistence may enhance the capabilities of terrorist organizations, the forces of CT are perpetually reinforcing the coercive iron cage. In business contexts, the formation of coercive isomorphism is the result of an explicit regulatory process: rule-making, monitoring, and sanctioning activities. Both government rules and forces from culture and society can impose standards on business organizations. For organizations in the organizational field, failure to conform to institutional expectations can have profound implications for the organization's legitimacy, ultimately leading to loss of tax-exempt status, loss of grants and contracts, or involuntary dissolution. Under the pressure of legitimacy and specific circumstances, business organizations may choose to operate primarily for legitimacy rather than for-profit.

In terrorism and CT contexts, such a process of coercive isomorphism is not directly applicable to terrorist organizations. The traditional binary relationship between government and business is challenging to apply to “autonomous” (Helfstein, 2009) organizations such as terrorist organizations. The claims of legitimacy of business organizations do not coincide with the claims of terrorist organizations. Nevertheless, this does not mean that the institutional theory fails to explain the isomorphism of terrorist organizations' behavior in the dimension of coercive isomorphism. The demands of terrorist organizations are more political than economic (Jongman, 2005). As far as legitimacy is concerned, referring to the work of Zarakol (2011), this article argues that the “legitimacy” of a terrorist organization is more likely to come from (a) Recognition by peer organizations, internal members; (b) The support of backers, potential militants; (c) The relative stability of political claims. If the government-business organization represents a healthy and mutually beneficial relationship, the government-terrorist organization relationship is characterized by hostility and unilateral sanctions.

The formation of terrorist organizations comes from the root cause of terrorism, which usually includes formal support from anti-government, anti-social forces, and deep-seated informal contradictions in society, culture, economy, and politics. As a group with political agenda, terrorist organizations usually aim to change the power base of a target state, thus creating a hostile binary relationship with the government. However, in the face of the government, the representative of the state's authority, terrorist groups have a dangerous role to play in the war on terror, especially when the government is relatively strong. In this case, it is bound to face a “legitimacy crisis” That is to say, in such a relationship, the terrorist organization is subjected to coercive sanctions that are far more powerful than those imposed on ordinary businesses, no longer bound by laws or rules but by military and police forces.

When terrorist organizations operate primarily in regions where the countries are stable and powerful, they are always subject to more stringent repressive responses. Sovereign governments and the corresponding authorities have always been able to respond promptly, for example, by intervening immediately when attacks or hotspots of attacks occur and by adopting long-term and proactive prevention strategies to address root causes of terrorism further. In this scenario, terrorist organizations would gradually weaken while losing some survival space. As criminals with less bargaining power, terrorist organizations will choose the most logical way to respond to sanctions, and the low-cost, more lethal, and easier-to-use bombings are common to terrorist organizations than other attacks that require sophisticated planning, surveillance, and staffing. Moreover, in situations where governments are weak or even disintegrating, as in Iraq and Afghanistan, terrorist groups often have more bargaining power due to the growth of anti-government forces. In such cases, hostage-taking, aircraft hijacking, and other terrorist attack methods may bring more economic and political gains, and bombing attacks may no longer be their favorite option.

Therefore, the effect of duration is not absolute, and when the government is stable, even long-dormant terrorist groups can hardly have forces hostage to bargaining with the government. Thus, this article proposes the following moderating effect:

**H3**: The negative effect of duration is attenuated when terrorist organizations carry out terrorist attacks mainly in regions with strong governments, which means there is a positive moderating effect.

**Figure 3:**
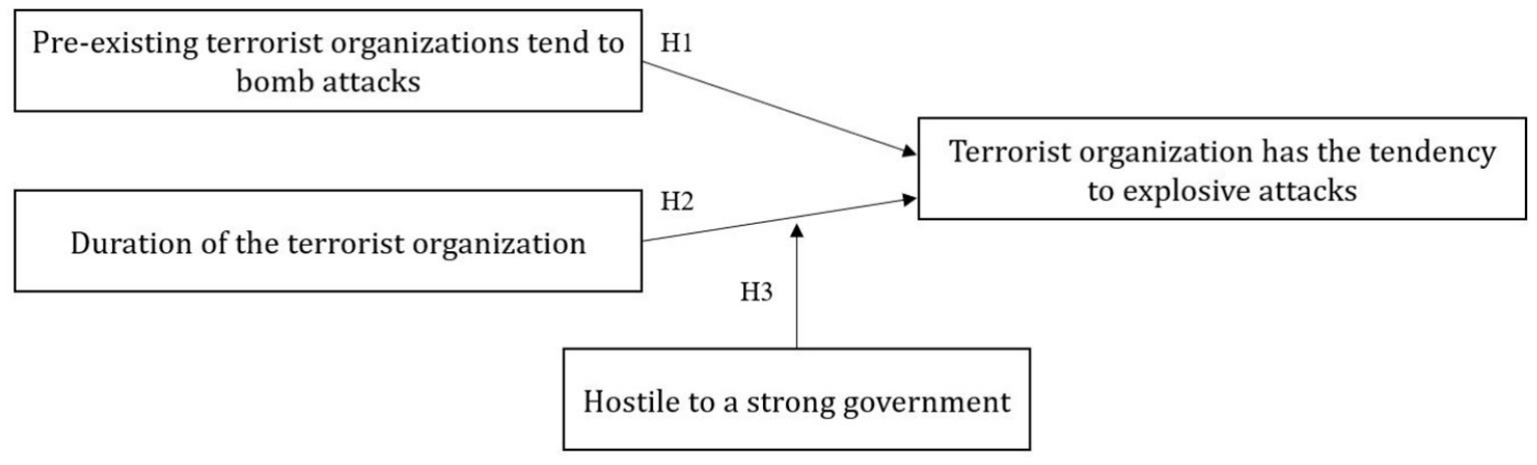
A hypothetical model of the terrorist organizations to conduct explosive attacks.

In summary, the conceptual explanatory model of the tendency of terrorist organizations to explosive attacks from the perspective of an institutional theory constructed in this article is shown in Figure 3.

## 4. Materials and Methods

### 4.1. Data Sources

The data used in this study are from the GTD database. GTD is currently one of the most complete databases of terrorist attacks globally, mainly collected and maintained by the National Consortium for the Study of Terrorism and Responses to Terrorism (START) in the United States (START, 2021). Till the end of 2017, GTD has collected more than 180,000 terrorist attacks in 12 regions of more than 200 countries over half a century, and all the incidents are indexed with variable “eventID.” About 98,000 cases with specific terrorist organizations have been identified among all records, and more than 3,500 terrorist organizations have been recorded. In addition, the GTD records detailed information about the time, method of attack, target, attacked country, etc., of each case, which matches the needs of this study.

In order to transform the data into a terrorist organization indexing approach suitable for this problem, this study follows such steps for data collection: (1) Excluding incomplete data due to loss of START files in 1993. (2) Excluding cases marked as “Unknown” in the “gname” column. (3) Excluding terrorist organizations with no more than one attack, cause these groups' attack tendency cannot be calculated. Since most terrorist organizations do not last long and often fall within a short period, it is challenging to obtain panel data on a year-by-year basis. Therefore, in order to ensure the completeness of the data, this article finally obtains a total of 1825 terrorist organizations' cross-sectional data spanning half a century (*N* = 1,825).

### 4.2. Dependent Variables

According to the conceptual explanatory model in Figure 3, the concept of the dependent variable is “Terrorist organization tends explosive attack.” For a given terrorist organization, this study measures it using a 0-1 binary variable. For attacks launched by a specific terrorist organization, if explosive attacks are the most frequent, more than other types in GTD, then the dependent variable is marked with “1,” and if explosive attacks are not the most, then marked with “0.” It is worth noting that if an organization has the same total counts of two attack types, then this study assumes that the organization tends toward the first occurred type.

### 4.3. Independent Variables

“Pre-existing terrorist organizations tend to explosive attacks” is the independent variable for H1. If a specific terrorist organization operates in the same country where the later organization specified in the dependent variable is primarily active, and before the latter's first activity, it has already been carrying out terrorist attacks, this specific organization is considered to be a “pre-existing” group. It is worth noting that there is usually more than one pre-existing terrorist organization for most organizations considered in the dependent variable. Here, the “pre-existing terrorist organization” is subject to temporal and geographical constraints to ensure logical integrity and constraining chronological relations to prevent the endogenous problems of reverse causation. For each certain terrorist organization mentioned in the dependent variable, once the collection of pre-existing groups has been identified, the tendency to explosive attack can be calculated for each pre-existing organization based on the principle of “whether the explosive attack is the most numerous among the eight types of terrorist attack.” Finally, if the proportion of pre-existing terrorist organizations with a tendency to explosive exceeds 50%, it is considered that the value of this independent variable is “1,” otherwise, it is “0.” It is important to note that if a terrorist organization appears for the first time in a specific country, there is no pre-existing group for it, which means the independent variable is set to “0,” and there is no object to imitate.

The concept “Duration of the terrorist organization” is firstly calculated as a continuous value accurate to year-level, with the results for a specific terrorist organization being the difference between the “iyear” value of the first attack and the last attack. Namely, the margin between the years. Secondly, considering that other explanatory variables are categorical variables and that scholars such as Yang and Zhou (2005) treat the age of firms in segments when applying institutional theory to the study of organizational behavior, this study coded the terrorist organization duration categorization as follows: “1” for terrorist organizations whose duration year margin is 0 in the first step, which means these groups are destroyed in a short period of time; “2” for organizations of one year's duration; the code is “3” for a duration of two years; “4” for three to five years; and “5” for more than six years. Ultimately, long-standing and notorious organizations such as the Taliban and Al-Qaida are in the category “5.”

### 4.4. Moderating Variable

The concept “Hostile to a strong government” is the moderating variable for H3. In order to ensure the reasonableness of the measure, this study first uses the “A-Government Stability” s from the International Country Risk Guide (ICRG) (Howell, 2011; PRS.Group, 2021) to measure the strength of governments around the world. The ICRG evaluates each country's annual “A-Government Stability” rating based on three indicators: government unity, legislative strength, and popular support. The highest score for each indicator is 4, with a higher score indicating a stronger government. The average government stability for all countries since 1984 is 7.57, using this s as a boundary to classify high and low groups, a total of 67 countries are relatively stronger, including China (8.83), Canada (8.05), Finland (8.05), and the United States (8.54). Secondly, since the records of GTD started in 1970 and ICRG in 1984, the ICRG lacks early records from regions such as the former Soviet Union and parts of Africa. In order to avoid large deviations from the facts, this study relaxes the precision of the judgment of “whether there is a strong government” to the “region” level in GTD. Ultimately, “East Asia,” “Western Europe,” “North America,” and “Australia and Oceania” are considered regions with relatively strong governments. For a given terrorist organization, the value of this dependent variable is “1,” if its primary activity area is one of the above four. Further, the “Central America and the Caribbean,” “South America,” “South-East Asia,” “South Asia,” “Central Asia,” Eastern Europe,” “The Middle East and North Africa,” “Sub-Saharan Africa” are considered to be regions with relatively weak governments. If a terrorist organization mainly attack countries in these areas, then the independent variable is “0.” The countries and regions in which terrorist organizations are mainly active are the plurals of the two corresponding records corresponding in the GTD.

### 4.5. Control Variables

In addition to the above variables, since there are few empirical studies on the behavior of terrorist organizations, this study proposes the following six control variables concerning the control of organizational size and structure by Yang and Zhou (2005): (a) “Whether the terrorist organization has a background of transnational attacks,” if a terrorist organization has carried out attacks in multiple countries and regions according to GTD records, then the code is 1, and vice versa is 0. (b) “Success rate of explosive attacks of the terrorist organization.” (c) “Success rate of explosive attacks by pre-existing terrorist organizations,” which is calculated as the proportion of terrorist attacks that are considered “successful” by GTD. Both the control variables (b) and (c) take into account that a higher success rate may impact the tendency to explosive attacks. (d) In order to prevent the effects of cooperation on organizational similarity in the normative isomorphism perspective, “whether a terrorist organization cooperates with an organization which has explosive tendency” is selected as the fourth control variable. If the particular terrorist organization is present in one or more of the attacks recorded by the GTD together with other explosive-prone organizations, the code is 1, and 0 otherwise. (e) Taking into account the impact of warfare on the propensity to bomb, for example, through the relative availability of bomb-making materials, it is crucial to take into account “Whether terrorist organizations operate mainly in relatively high-risk areas.” If a terrorist organization is mainly active in the Middle East and North Africa, Southeast Asia, South Asia, and Central Asia, the code is 1, and the opposite is 0. (f) Similar to the transnational context, in order to further control the size of the organization, this study controls “The number of attacks by terrorist organizations” and codes the number of terrorist attacks from 0–10, 11–100, 101–500, 501–1000 and above 1001 to a scale of 1–5.

A brief identification and explanation of all the above variables in this study are shown in Table 2.

**Table 2:** Variables identification and definition.

**Variable**	**Meaning**	**Variable ID**	**Variable format**
Dependent	Terrorist organization has the tendency to explosive attack	TENDENCY	Binary
Independent	Pre-existing terrorist organizations tend to explosive attacks	EX_TEND	Binary
	Duration of the terrorist organization	AGE	1-5 ordered categorical
Moderation	Hostile to a strong government	ANTI	Binary
Controls	Whether the terrorist organization has a background of transnational attacks	TRANS	Binary
	Success rate of explosive attacks of the terrorist organization	SUCCESS	Continuous
	Success rate of explosive attacks by pre-existing terrorist organizations	EX_ SUCCESS	Continuous
	whether a terrorist organization cooperates with an organization which has explosive tendency	COOP	Binary
	Whether terrorist organizations operate mainly in relatively high-risk areas	R_AREA	Binary
	The number of attacks by terrorist organizations	FREQ	1-5 ordered categorical

## 5. Results

### 5.1. Descriptive Statistics and Multi-Collinearity Analysis

This study uses the logistic regression method to analyze 1,825 cross-sectional data from the GTD. Table 3 shows the results of descriptive statistics and multi-collinearity analysis. As the variables are mainly categorical variables, Spearman's correlation test was used. From the table: (a) The data for the remaining variables are well distributed, except for the COOP with a mean of 0.07, which is heavily skewed to the left. (b) EX_TEND was significantly correlated with TENDENCY, with a correlation coefficient of 0.163 (*p* < 0.01), ANTI is also significantly correlated with TENDENCY, with a coefficient of 0.105 (*p* < 0.01), H1 and H2 are initially confirmed. (c) AGE was negatively correlated with TENDENCY, but the correlation coefficient −0.016 was not significant (*p* > 0.05), the H3 needs further examination. (d) The variance inflation factors (VIF) of the main variables are between 1.082 and 2.254, smaller than the criterion for multi-collinearity discrimination (<5). This indicates that the problem of multi-collinearity is not serious, but in order to ensure the experimental rigor, the correlation coefficients greater than 0.7 are excluded from the regression analysis. This means SUCCESS and EX_SUCCESS are excluded from the regression analysis.

**Table 3:** Descriptive statistics and multi-collinearity analysis of variables.

**Variable**	**Min**	**Max**	**Mean**	**Std**.	**VIF**	**1**	**2**	**3**	**4**	**5**	**6**	**7**	**8**	**9**	**10**
TENDENCY	0	1	0.45	0.498	\	1.000									
EX_TEND	0	1	0.38	0.486	2.154	0.163^**^	1.000								
ANTI	0	1	0.29	0.454	1.582	0.105^**^	0.247^**^	1.000							
AGE	1	5	3.025	1.687	1.471	−0.016	-0.087^**^	−0.101^**^	1.000						
TRANS	0	1	0.28	0.449	1.337	−0.009	-0.059^*^	−0.046^*^	0.452^**^	1.000					
SUCCESS	0	1	0.367	0.355	1.082	0.807^**^	0.174^**^	0.072^**^	−0.040	−0.023	1.000				
EX_SUCCESS	0	1	0.341	0.193	2.254	0.180^**^	0.774^**^	0.229^**^	−0.106^**^	−0.129^**^	0.215^**^	1.000			
COOP	0	1	0.07	0.255	1.122	0.067^**^	0.053^*^	−0.133^**^	0.086^**^	0.000	0.073^**^	0.083^**^	1.000		
R_AREA	0	1	0.418	0.493	1.568	0.055^*^	−0.047^*^	−0.541^**^	0.093^**^	0.005	0.100^**^	0.087^**^	0.202^**^	1.000	
FREQ	1	5	1.399	0.712	1.456	0.096^**^	−0.035	−0.091^**^	0.516^**^	0.363^**^	0.069^**^	−0.095^**^	0.240^**^	0.087^**^	1.000

*N = 1,825*;

* *p < 0.05*;

** *p < 0.01*.

### 5.2. Logistic Regression Analysis Method for Cross-Sectional Data

Logistic regression analysis is applicable to problem scenarios where the dependent variable is a binary variable, and does not emphasize whether the variables are normally distributed, thus it is adopted for hypothesis testing in this article. The logistic regression method is as shown in Equation (1):


(1) $$\begin{array}{l} Logistic\left(p\right)=ln\frac{p}{1-p}=\beta_{0}+\beta_{1}x_{1}+\beta_{2}x_{2}+...+\beta_{n}x_{n}+\varepsilon \end{array}$$

where *p* = *p*_(*y* = 1)_ represents the probability that the dependent variable *y* is equal to 1, which means the probability that a terrorist organization has a tendency to exposion attack. $\frac{p}{1-p}$ is the odds of experiencing an event (odds). β_*i*(*i*=0,1,2...*n*)_ is the regression coefficient of the variable, β_0_ is the constant, ε is the random error. *x*_*i*(*i*=0,1,2......*n*)_ are other variables, and *n* is the number of variables. The logistic model uses the maximum likelihood method for parameter estimation.

### 5.3. Results of Logistic Regression Analysis

This article is divided into five models for logistic regression analysis. Model 0 is the base model with only four control variables added: TRANS, COOP, R_AREA. FREQ, as a comparative baseline for subsequent models. Models 1-2, respectively, add EX_TEND and AGE as independent variables to test H1-H2. Model 3 adds a moderating effect, which means the product term of ANTI and AGE. Model 4 is a full-variable model used to reflect whether the results are robust or not tentatively.

In Model 1 of Table 4, the EX_TEND coefficient of 0.690 is significantly positive (*p* < 0.001), indicating that the H1 Confirmation that the “tendency of pre-existing terrorist organizations to explosive attacks” does have a positive effect on the tendency of later ones. From Equation (1), the primary effect size of H1 is *Exp*(*B*_1) = *e*(0.690) = 1.994, which means if the tendency to the explosive attack of a pre-existing terrorist organization changes from 0 to 1 (the independent variable increases by 1), and the odds of the later terrorist organizations to form explosive tendency are 1.994 times greater. The above analysis shows that the mimetic isomorphism effect based on institutional theory significantly impacts the formation of terrorist group explosive tendencies. In model 2, the coefficient of AGE is -0.093 (*p* < 0.01), which means H2 is confirmed and “Duration of the terrorist organization” does diminish its tendency to explosive attacks. The size of this negative effect is *Exp*(*B*_3) = 0.912, which indicates that once the duration of a terrorist organization increases by one level, the probability of forming explosive tendency is only 91.2%, which is about 10% lower than the original probability. These two hypotheses were confirmed in the separate models and in the full-variable model 4, where the coefficients are significant, and the sign directions do not change, which to some extent indicates the robustness of the results. The regression result of the moderating effect of ANTI*AGE in H3 is shown in models 3 and 4, which reveals the coefficients of interaction are both respectively positive: 0.155 (*p* < 0.05) and 0.137 (*p* < 0.05). This result confirms H3 and shows that the ANTI as a moderating variable significantly reduces the negative effect of terrorist organization duration on the formation of explosive tendency.

**Table 4:** Results of logistic regression analysis.

**Variable**		**Model 0**	**Model 1**	**Model 2**	**Model 3**	**Model 4**
Controls	TRANS	−0.240^*^ (0.116)	−0.204^+^ (0.118)	−0.135 (0.123)	−0.122 (0.125)	−0.108 (0.126)
	COOP	0.252 (0.196)	0.173 (0.199)	0.249 (0.197)	0.275 (0.198)	0.209 (0.201)
	R_AREA	0.163^+^ (0.098)	0.209^*^ (0.100)	0.184^+^ (0.099)	0.652^***^ (0.121)	0.599^***^ (0.122)
	FREQ	0.322^***^ (0.076)	0.335^***^ (0.078)	0.403^***^ (0.083)	0.437^***^ (0.084)	0.431^***^ (0.085)
Independent	EX_TEND		0.690^***^ (0.099)			0.523^***^ (0.103)
	AGE			-0.093^**^ (0.034)	−0.136^***^ (0.040)	−0.121^**^ (0.040)
	ANTI				0.468^*^ (0.219)	0.353 (0.222)
Interaction	AGE ^*^ ANTI				0.155^*^ (0.063)	0.137^*^ (0.063)
Intercept	Constant	−0.676^***^ (0.113)	−0.987^***^ (0.124)	−0.548^***^ (0.123)	−0.933^***^ (0.156)	−1.103^***^ (0.161)
*N*		1,825	1,825	1,825	1,825	1,825

+ *p < 0.1*,

* *p < 0.05*,

** *p < 0.01*,

*** *p < 0.001*.

*Standard errors of the coefficient estimates are in parentheses*.

### 5.4. Robustness Test

In order to ensure the reliability of the above results, this study chose another measure of the dependent variable to test the robustness of the model. In section 3, the concept of “whether a terrorist organization has the tendency to explosive attack” as the dependent variable is measured based on “whether the number of bombing/explosive is the most among the eight methods.” In this section, the measure is changed to “whether the number of bombing attacks exceeds 50% of the total number” during the robustness test, which makes the measure stricter, while the dependent variable remains a binary variable. Robustness tests were conducted by replacing variables of the same nature: the models R0-R4 are based on the original logistic regression model, with the dependent variable is replaced by the new measure. The Robustness test results are shown in Table 5. The three confirmed hypotheses also hold in the robustness test. The EX_TEND coefficient is 0.816 (*p* < 0.001), and the effect size is *Exp*(*B*_1) = 2.262. The AGE coefficient is −0.164 (*p* < 0.001), negative effect *Exp*(*B*_3)=0.849. The coefficients of the two main effects remain significant and increase in size, further illustrating the reliability of the hypothesis. The moderating effect is also confirmed, with an intersection coefficient of 0.175 (*p* < 0.01) for model R3 in Table 5. The above findings are all valid for the full variables model R4. The above analysis shows that the model passes the robustness test, and the results are reliable.

**Table 5:** Robustness test results.

**Variable**		**Model R0**	**Model R1**	**Model R2**	**Model R3**	**Model R4**
Controls	TRANS	−0.271^*^ (0.122)	−0.231^+^ (0.125)	−0.084 (0.130)	−0.072 (0.133)	−0.050 (0.134)
	COOP	0.402^*^ (0.197)	0.311 (0.201)	0.399^*^ (0.198)	0.436^*^ (0.201)	0.360^+^ (0.203)
	R_AREA	0.033 (0.102)	0.086 (0.105)	0.069 (0.103)	0.611^***^ (0.131)	0.543^***^ (0.133)
	FREQ	0.021 (0.079)	0.027 (0.080)	0.161^+^ (0.084)	0.194^*^ (0.087)	0.181^*^ (0.087)
Independent	EX_TEND		0.816^***^ (0.102)			0.634^***^ (0.106)
	AGE			−0.164^**^		
(0.036)	−0.221^***^ (0.043)	−0.203^***^ (0.043)				
	ANTI				0.512^*^ (0.224)	0.373 (0.229)
Interaction	AGE ^*^ ANTI				0.175^**^ (0.064)	0.152^*^ (0.065)
Intercept	Constant	−0.647^***^ (0.116)	−1.017^***^ (0.128)	−0.421^***^		
(0.125)	−0.846^***^ (0.162)	−1.050^***^ (0.168)				
N		1,825	1,825	1,825	1,825	1,825

+ *p < 0.1*,

* *p < 0.05*,

** *p < 0.01*,

*** *p < 0.001*.

*Standard errors of the coefficient estimates are in parentheses*.

**Figure 4:**
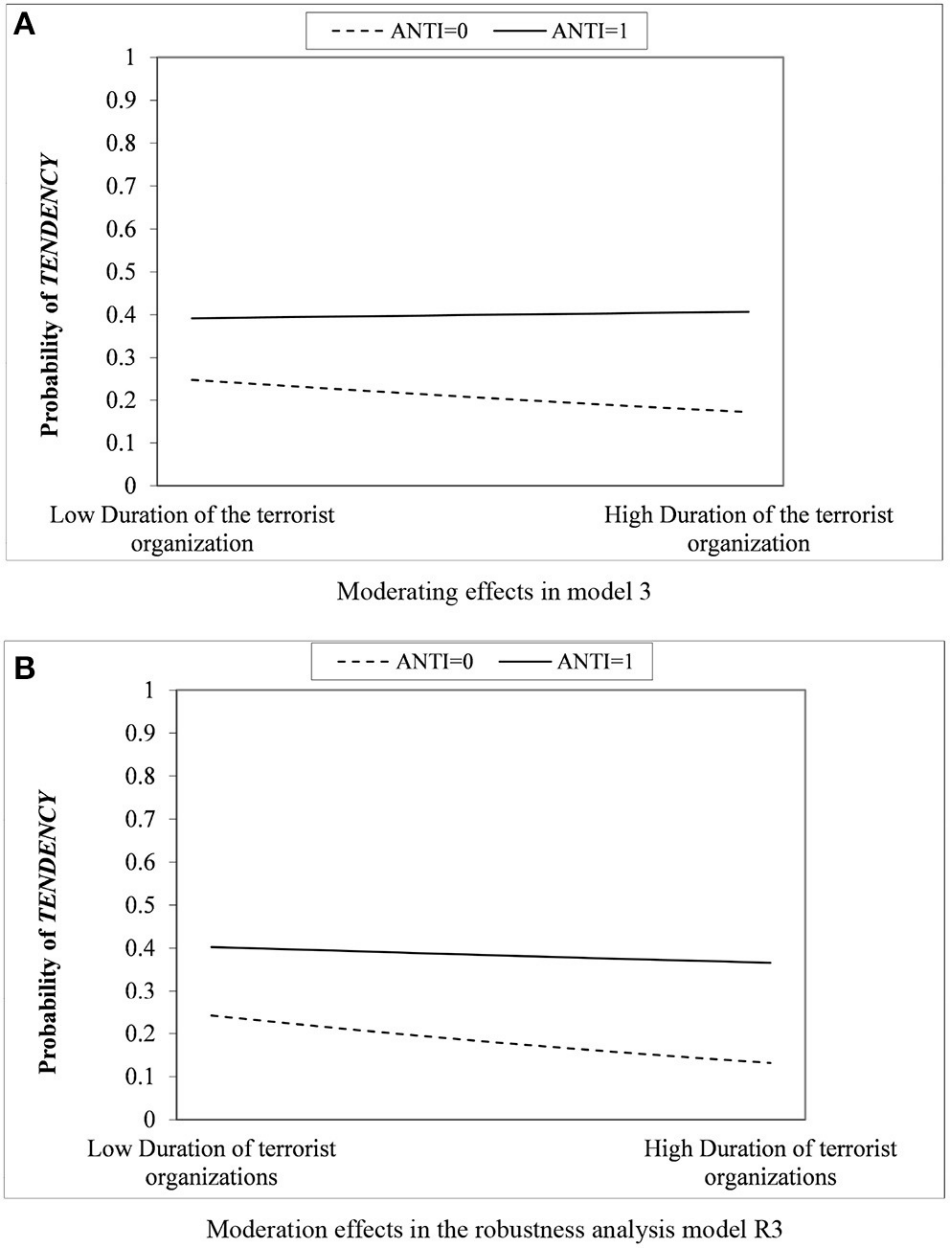
Coercive isomorphic moderating effects in terrorist duration-explosive tendency. **(A)** Moderating effects in model 3. **(B)** Moderation effects in the robustness analysis model R3.

This study further graphically analyzes the moderating effects of model 3 in Table 4 and model R3 in Table 5 to understand better the role of coercive and mimetic isomorphisms in forming terrorist organizations' explosive tendencies. The horizontal axis of the figure shows the high and low groups of the duration “AGE,” using the mean plus or minus one standard deviation as the high-low grouping boundary, with the vertical axis being the probability that a terrorist organization tends to explosive attacks. As shown in Figure 4A, where the dependent variable is “whether terrorist organizations use explosive attacks the most of the eight methods,” the dashed regulatory effect group was clearly above the Solid line unmodulated effects group. This phenomenon suggests that terrorist organizations operating in areas with a stable government are more prone to explosive attacks than those operating in areas with a weak government. At the same time, the probability of explosive tendency decreases significantly with increasing duration of terrorist organizations in the solid-line group without adjustment effect, which once again confirms the negative effect of terrorist organization duration on the tendency in H3. The negative effect of the duration is significantly mitigated in the dashed group corresponding to the moderating effect, and the slope is relatively flat. Figure 4B shows the moderating effect in the robustness analysis, and again, terrorist groups under the moderating effect are significantly more likely to prefer explosive attacks regardless of their duration. In summary, the four hypotheses, three main effects, and one moderating effect proposed in this article are all confirmed.

## 6. Discussion

As the three hypotheses proposed in this study are validated, we will highlight how the analysis of explosive attack tendency isomorphism within institutional theory's perspective can implicate CT practices. Meanwhile, we will also explore the limitations of the study.

The influence of pre-existing terrorist organizations on the explosive tendency of subsequent terrorist organizations operating primarily in the same countries is intense, and the mimetic isomorphic shows significant effects. It is crucial for states, security personnel and CT experts to interrupt this contagion-like process. The destruction of the learnable attack experience is one of the critical means. For example, when it is found that pirates want to learn from previous successful experience and continue operating in locations where there have been results, a successful rescue, to destroy the “this place is conducive to attack” experience, it can greatly ameliorate the security situation (Di Salvatore, 2018). This is helpful situational crime prevention (SCP) approach, which means adopting situational measures to reduce the chances of offending to prevent specific types of incidents (Clarke, 2013). The experiences formed from one successful attack consist of elements such as the target's vulnerability in this incident, the method of weapon usages, the results, etc. Strengthening protection for similar vulnerable targets, increasing the difficulty of acquiring similar weapons, and reducing the spread of fear may be effective ways to reduce learning among terrorist organizations.

Although the effect is not as strong as mimetic isomorphism, normative and coercive isomorphism also significantly impact the similarity of terrorist organizations. And these two isomorphic processes dialectically reveal the relationship between terrorist organizations and their institutional cages. The longer a terrorist organization survives, the more capable it becomes, and the richer its tactics and targets. If the country in which it operates is strong enough, then it may also suppress this trend. How to go beyond merely suppressing the diverse capabilities of terrorist organizations to further stop terrorist organizations and terrorism? The first answer is to address the root causes of terrorism. This is a long-term program that requires improvements in all aspects of politics, economics, conflict, etc., that we have mentioned. Without a turbulent institutional environment, the citizens will lose their reason to radicalize, potential supporters will abandon their commitment to terrorism, and terrorist groups' delusions of becoming more professional will only get more ridiculous. On the other hand, when the root causes are addressed, a formed terrorist organization may continue to operate until it is entirely physically eliminated (Crenshaw, 1981). At this point, it must be coupled with the short-term approaches discussed in the analysis of mimetic isomorphism, which is also highlighted by Bjørgo (2013): protecting the vulnerable targets, disrupting the planned attacks, mitigating tragic consequences, reducing feedback from attacks to terrorist organizations, etc.

Overall, while the trend of terrorist attacks is still severe, the number of global terrorist attacks has increased in the last decade. But with the involvement of studies in the field of terrorism and CT, this dismal situation may be reversed. It is also hoped that this article can provide some modest theoretical contributions to public security.

There are two main limitations of this article. First, it is challenging to obtain panel data containing a relatively large number of organizations. While GTD contains very detailed data on global terrorist attacks, unfortunately, many terrorist attacks are hard to know the terrorists or terrorist organizations that perpetrated them, which creates limitations. Second, we have not addressed the issue of transnational terrorist organizations sufficiently. As the three hypotheses concern, the current study focuses primarily on the institutional environment posed by a single country, while the more complex situations faced by transnational organizations we have not adequately considered. In future research, we will further apply institutional theory to study transnational terrorist organizations' behavioral patterns.

## 7. Conclusion

This article analyzes and empirically investigates the explosive tendency of terrorist organizations from three perspectives: coercive, normative, and mimetic isomorphism, based on institutional theory. The results indicate that (a) Terrorist organizations will learn from the experience of pre-existing terrorist organizations and that this imitative behavior will contribute to the formation of explosive tendencies. (b) As the duration of a terrorist organization increases, its explosive tendency diminishes or disappears. This is due to the longer duration, which means the retention of strategic space for terrorist organizations, the weakness of governments, and increased bargaining power. At this point, multiple methods of a terrorist attack may be an option, and bombing is no longer the only choice. (c) Terrorist organizations often have little room for maneuver and little bargaining power when confronted with a strong government. This can lead to a preference for relatively more straightforward and more damaging explosive attacks when avenues such as hijacking and kidnapping are challenging to pursue. Thus, if terrorist groups operate primarily in strong government strongholds, their growth over time will not allow them to flourish. In the face of an intense government siege, explosive attacks are likely to remain at the of their propensity.

The above findings illustrate the applicability of the institutional theory in explaining the isomorphism of terrorist behavior, especially the convergence of attack methods. Moreover, they elucidate the role of coercive and normative isomorphic drivers in the isomorphism of terrorist behavior, which has received little attention in previous studies: unlike the isomorphic reasons why business organizations are subject to government regulation and industry norms, the claim of “legitimacy” of terrorist organizations is anti-government, the widespread use of bombings as a better bargaining tool for terrorist activities in order to survive in the general war on terror.

The study suggests that counter-terrorism approaches such as destroying the learnable experience of attacks, addressing the root causes of terrorism, and maintaining a strong government may be helpful in stopping increasing terrorist activities. These strategies imply popular support, unity, and coordination among all counter-terrorism departments, which firstly curbs the activities of all terrorist organizations and blocks the communication between them. Finally, with a strong government suppression, the existence of terrorist organizations will be relatively shorter, which will also make their strategic space narrow and their ability to do evil diminished. In addition, this research speculates that end-of-the-road terrorist organizations may be more tend to explosive attacks, and future research will attempt to examine the issue of terrorist attack prediction in conjunction with this analysis.

## Data Availability Statement

Publicly available datasets were analyzed in this study. This data can be found at: Global Terrorism Dtabase, https://www.start.umd.edu/gtd/.

## Author Contributions

All authors listed have made a substantial, direct, and intellectual contribution to the work and approved it for publication.

## Funding

This work was supported by the National Key Research and Development Program of China (Award numbers: 2018YFC0807500) and National Natural Science Foundation of China (Award numbers: 71974067 and 71821001).

## Conflict of Interest

The authors declare that the research was conducted in the absence of any commercial or financial relationships that could be construed as a potential conflict of interest.

## Publisher's Note

All claims expressed in this article are solely those of the authors and do not necessarily represent those of their affiliated organizations, or those of the publisher, the editors and the reviewers. Any product that may be evaluated in this article, or claim that may be made by its manufacturer, is not guaranteed or endorsed by the publisher.
